# Omalizumab therapy in a 13-year-old boy with severe persistent asthma and concomitant eosinophilic esophagitis

**DOI:** 10.1186/s13052-016-0243-x

**Published:** 2016-03-22

**Authors:** Stefania Arasi, Stefano Costa, Giuseppe Magazzù, Antonio Ieni, Giuseppe Crisafulli, Lucia Caminiti, Fernanda Chiera, Mario Vaccaro, Michele Miraglia Del Giudice, Giovanni Battista Pajno

**Affiliations:** Department of Pediatrics- Allergy Unit, University of Messina, Via Consolare Valeria- Gazzi, 98124 Messina, Italy; Department of Pediatrics- Gastroenterology Unit, University of Messina, Via Consolare Valeria- Gazzi, 98124 Messina, Italy; Department of Human Pathology, University of Messina, Via Consolare Valeria- Gazzi, 98124 Messina, Italy; Department of Dermatology, University of Messina, Via Consolare Valeria- Gazzi, 98124 Messina, Italy; Department of Pediatrics, II University of Naples, via De Crecchio 4, 80138 Naples, Italy

**Keywords:** Severe persistent asthma, Eosinophilic esophagitis, Topical steroids, Omalizumab

## Abstract

**Background:**

Eosinophilic esophagitis (EoE) has been defined as “asthma of the esophagus” for the large number of similarities between the two diseases. Omalizumab is an anti-Immunoglobulin E (IgE) antibody currently approved only in allergic IgE-mediated severe persistent uncontrolled asthma and in chronic spontaneous urticaria unresponsive to antihistamines, but it has been tried in other diseases, too.

**Case presentation:**

We present herein the case of a 13-year-old boy, affected from preschool age by severe chronic allergic asthma poorly controlled despite a generous long-term therapy, and, since he was 8 years old, by eosinophilic esophagitis, responsive to courses of strict elimination diet and semi-elemental diet, even if very burdensome for his quality of life.

At the age of 11.5 years, for inadequate asthma control, he started to receive therapy with omalizumab. After the first month and for the entire duration (18 months) of omalizumab treatment, asthma was well controlled, long-term conventional therapy was gradually withdrawn and lung- function improved. Concerning EoE, after an initial clinical but not histological remission during the first few months of treatment with omalizumab, the patient experienced an exacerbation of gastrointestinal symptoms. Therefore, he started treatment with topical steroids which was effective to improve gastrointestinal symptoms. However, EoE is still steroid-dependent. Currently, he continues both treatments: omalizumab for asthma and topical steroid for EoE.

**Conclusions:**

This case report confirms that omalizumab is an effective treatment in patients with severe persistent, uncontrolled asthma. On the other hand, in our patient it did not produce persistent improvement neither on symptoms nor on biopsy findings of EoE. The outcome of this case might indicate different pathogenic mechanism(s) of the two diseases.

## Background

Eosinophilic esophagitis (EoE) has been defined as “asthma of the esophagus” for the several similarities between the two diseases [1, 2]. The prevalence of both diseases is increasing over time –as other atopic conditions- and, they often coexist: asthma has been reported in higher percentage (up to 80 %) in subjects with EoE [3]. Both asthma and EoE are chronic immune-mediated and most likely antigen - driven conditions [2]. Food allergy often precedes asthma and it seems to be driving EoE, in children. In adult and teenagers, allergic sensitization to aeroallergens is often associated not only with asthma but also with EoE, worsening their prognosis [2]. Inflammation of mucosa and submucosa with a typical infiltration by eosinophils is hallmark of both conditions [2]. Allergen avoidance may improve symptoms in both diseases without curing them [2].

On the basis of similarities it is reasonable to realize that similarities “could mirror” also in therapy. Omalizumab is a humanized monoclonal anti-Immunoglobulin E (IgE) antibody currently approved only in allergic IgE-mediated severe persistent asthma and in chronic spontaneous urticaria unresponsive to antihistamines [4] but it has been tried as off-label treatment in other diseases [5], in particular in some isolated cases of EoE with different results as described in some previous reports [6–8]. We report the case of a 13-year-old boy, affected by severe chronic asthma inadequately controlled with high dose of inhaled corticosteroids and montelukast and EoE treated with omalizumab -for a longer period (18 months) than previous reports for EoE- with full remission of allergic asthma but not of eosinophlic esophagitis.

## Case presentation

A 13-year-old boy, affected by severe chronic asthma with abnormal lung function: a forced expiratory volume in 1 s (FEV_1_ < 80 %) of the predicted value before bronchodilation with β_2_ agonist: salbutamol. The first respiratory symptoms appeared when he was about two years old.

He had dust mites and olive pollen allergies -confirmed by skin prick tests (average diameter of wheal for Dermatophagoides Pteronyssinus, Dermatophagoides Farinae, and Olive pollen: 9, 7, and 5 mm, respectively) and specific IgE levels (100, 82 and 68 IU/ml, respectively) and high total IgE (1003 IU/ml) levels with normal eosinophil count (110/mmc).

**Fig. 1:**
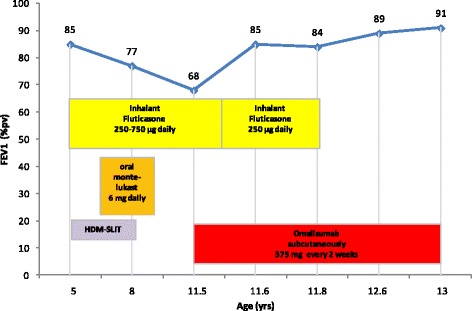
FEV_1_ (% predictive value) longitudinal profile in our patient. Changes in forced respiratory volume in 1 s (FEV_1_) over time are shown for different treatments. FEV_1_ significantly improved during omalizumab therapy. The most representative detections are represented

He performed long-term anti-asthma therapy with high-dose inhaled corticosteroids (fluticasone up to 750 μg/day), also associated with a leukotriene receptor antagonist (montelukast) and sublingual specific immunotherapy (SLIT) with dust mite extract (since he was 5 years old for 3 years continuously) with poor disease control (without any precise seasonal correlation) and reduction of FEV_1_ according to GINA guidelines (Fig. 1) (http://www.ginasthma.org/documents/1/Pocket-Guide-for-Asthma-Management-and-prevention).

**Fig. 2:**
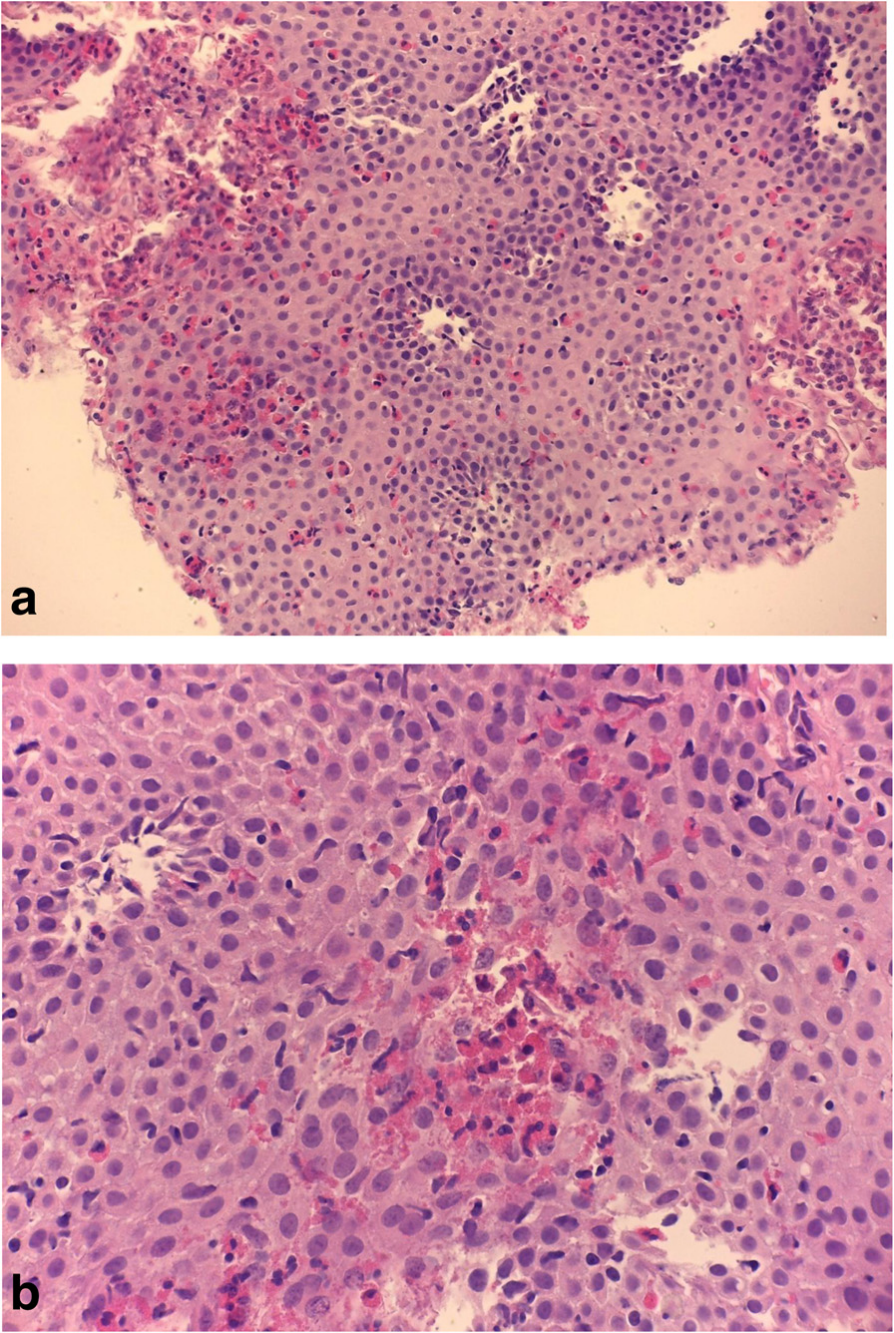
Esophageal histology. An evident eosinophilic cellular infiltrate was encountered in the esophageal epithelium; intercellular oedema as well as basal zone hyperplasia were also appreciable (**a**) [Haematoxylin and Eosin staining (H&E), original magnification x200]; sometimes eosinophils were organized in micro-abscesses (**b**) (H&E, original magnification x 400)

When he was 8 years old, for the appearance of gastrointestinal symptoms (nausea, vomiting, heartburn), after endoscopy-biopsy of the upper digestive tract, eosinophilic esophagitis was diagnosed (Fig. 2a and b).

The prick tests were positive to milk (average diameter of wheal for milk extract 8 mm; α-lactoalbumin 10 mm; β-lactoglobulin 7 mm; casein 6 mm; Prick by prick with fresh milk 10 mm) and soy (5 mm).

He was placed on semi-elemental diet with benefit on esophageal symptoms and a strict food allergens (milk, dairy products and soy) avoidance, and histology (performed after 3 months since the beginning of each therapy, respectively at 9.3 and 10 years of age) but with heavy burden on his quality of life. Symptomatic response to therapy (improvement/impairment/no change) was assessed evaluating changes of the patient’s answers concerning the following items: frequency of trouble swallowing; duration of trouble swallowing (≥/< 5 min); pain when swallowing. Furthermore, a visual dysphagia question addressed the severity of dysphagia when consuming food of 8 distinct consistencies. Behavioural adaptations (avoidance, modification, and slow eating of various foods) also were assessed in the context of consuming 8 distinct food consistencies [9]. Afterwards, courses of topical steroid therapy (oral budesonide) were also carried out with symptomatic and histological efficacy. The latter was detected after the second (at 10.6 years of age), and the sixth run (under omalizumab, at 12.6 years). However, the effects (on both symptoms and histology) were transient and the treatment was sometimes complicated by oral candidiasis, for which the therapy was interrupted and antimycotic treatment was performed by patient.

At the age of 11.5 years, for inadequate asthma control (in the last 2 months he presented daytime symptoms 3 to 4 times/week, some night waking due to asthma, reliever needed 2 to 3 times/week, limitation of his activities- e.g. going upstairs- due to asthma) (http://www.ginasthma.org/documents/1/Pocket-Guide-for-Asthma-Management-and-prevention), he started to receive therapy with omalizumab. According to technical data sheet, the dose and frequency of dosing were guided by a nomogram that is derived from the total serum IgE level and the body mass index (375 mg subcutaneously every 2 weeks). After the first month of treatment, conventional long-term therapy for asthma was reduced up to the suspension (at the 4^th^ month of omalizumab therapy), with an improvement both in asthma symptoms and in spirometric parameters for the entire duration of treatment (18 months) (Fig. 1). Broncodilatator reversibility test remained positive over time, before and after omalizumab therapy, with a significant increase in FEV_1_ after bronchodilator (salbutamol) indicating a reversible airflow obstruction and supports the diagnosis of asthma (http://www.ginasthma.org/documents/1/Pocket-Guide-for-Asthma-Management-and-prevention).

Regarding EoE, during the first three months of treatment the patient presented in clinical remission even if not accompanied by histological remission (endoscopy performed at the 3rd months of treatment). Afterwards, he experienced an exacerbation of gastrointestinal symptoms with confirmation of eosinophilic infiltrates in the esophageal mucosa (endoscopy at the 7^th^ month of treatment). Therefore, he performed treatment with topical steroids (galenical oral viscous budesonide) with improvement of nausea and vomiting. In detail, we used a product prepared by the chemist in the laboratory of our pharmacy. The product was made mixing a vial of budesonide (1 mg/2 ml) with 5 g of sweetener (2.5 g of aspartame and 2.5 g dextrose) [10, 11].

On the other hand, at the 18th month of omalizumab therapy the patient continues to have a good asthma control according to GINA assessment (http://www.ginasthma.org/documents/1/Pocket-Guide-for-Asthma-Management-and-prevention) without other anti-asthmatic therapy and his EoE is steroid-dependent. He is going on treatment with omalizumab s.c. and viscous budesonide per os.

## Discussion

The patient, with severe respiratory and esophageal symptoms, had a quality of life seriously compromised by his allergic diseases. Despite a heavy long-term treatment with high dose of inhalant steroids combined antileukotriene, his asthma remained poorly controlled; the semi-elemental diet and the strict food avoidance of milk and dairy products, although had provided an improvement, though transient, in esophageal symptoms, were very burdensome for the patient.

Omalizumab was effective therapy for severe persistent asthma in our patient, according to its conventional indication, but it did not produce persistent clinical improvement nor endoscopic and histological changes of his EoE.

Few cases of EoE treated with omalizumab (*n* = 2 [6], *n* = 9 [7], *n* = 15 [8]) have been reported in literature.

Authors of the first two studies described improvement in symptoms without a reduction of oesophageal eosinophils in all patients treated with omalizumab and food avoidance [6, 7]. The third one, a pilot study, open label, on 15 patients treated with omalizumab for 12 weeks, maintaining dietary restriction, found a histological and clinical improvement only in 33 % of the patients (subjects with low peripheral blood absolute eosinophil count) [8].

In our patient, different than other cases previously reported, no dietary restriction was maintained to verify the efficacy of the only treatment with omalizumab without risk of confounding. Dietary restriction is one of the main treatment options in patients with EoE and food allergy. Therefore, in the previous studies it is likely a possible overlap of effects of two treatments (food avoidance plus omalizumab).

This is only a single clinical case. However, considering the limitations of assumptions based on it, the therapeutic failure of omalizumab in EoE of our patient might highlight that omalizumab probably does not work on allergen-specific IgE-mediated pathogenic mechanism of EoE, or at least not mainly. In EoE allergic sensitization drives the formation of allergen-specific both IgE and T cells and they potentially have independent roles in the underlying disease pathogenesis [12]. Therefore, our data indicate that asthma and EoE, even sharing a similar allergenic features, are likely mantained by different pathogenic mechanisms, which need to be better understood.

In literature a unique case of EoE induced by SLIT to pollens is described. [13] A seasonal and geographical pattern of EoE which may be compatible with increased airborne allergen was reported, too [14, 15].

Cautiously, considering the literature paucity about a possible correlation between EoE and SLIT, it might be hypothesized that, likely, sublingual dust mite immunotherapy might cause or exacerbate EoE by exposing the esophagus to host dust mites allergens (HDM) every day. However, investigations are needed.

Notwithstanding, in our patient, esophageal symptoms were correlated temporally to food allergen-exposure and not to airborne (and in particular to HDM) exposure (esophageal symptoms appeared about three months after the end of SLIT to HDM).

We are aware of the limitations of assumptions based on one case only, but we report our experience because in our knowledge this is the first case of EoE treated with omalizumab for so a long period (18 months) and it may help to elucidate the pathophysiology of EoE and to select patients affected with EoE that could benefit of anti-IgE treatment. In the recent pilot study [8] omalizumab –induced remission of EoE was limited to subjects with low peripheral blood eosinophil count. Instead, our patient, despite a normal-low eosinophilic serum count before and during the entire duration of treatment, did not respond to omalizumab.

## Conclusions

In conclusion, the Th2 cytokines, IL-13 and IL-4, remain, currently, the most promising antieosinophil targets also in EoE and more studies are required in order to clarify the possibility of new therapeutic approaches such as omalizumab for EoE management.

The present case report confirms that omalizumab is an effective treatment in patients with severe persistent, uncontrolled asthma. On the other hand, in our patient it did not produce persistent improvement neither on symptoms nor on biopsy findings of EoE. The outcome of this case might indicate different pathogenic mechanism(s) of the two diseases. Well-designed and large studies are needed.

### Consent

Written informed consent was obtained from the patient for publication of this Case report and any accompanying images. A copy of the written consent is available for review by the Editor-in-Chief of this journal.

## Abbreviations

EoE: eosinophilic esophagitis
FEV_1_: forced expiratory volume in 1 second
H&E: haematoxylin and Eosin
HPF: high power fields
IgE: immunoglobulin E
